# Impact of low iodine density tumor area ratio on the local control of non-small cell lung cancer through stereotactic body radiotherapy

**DOI:** 10.1093/jrr/rrab015

**Published:** 2021-04-05

**Authors:** Mitsuki Tanaka, Ichise Koji, Ichitaro Fujioka, Mariko Sato, Katsumi Hirose, Hideo Kawaguchi, Yoshiomi Hatayama, Yoshihiro Takai, Eiki Tsushima, Masahiko Aoki

**Affiliations:** Department of Radiation Oncology, Graduate School of Medicine, Hirosaki University, 5 Zaifu-cho, Hirosaki, Aomori, 036-8216 Japan; Department of Radiation Oncology, Graduate School of Medicine, Hirosaki University, 5 Zaifu-cho, Hirosaki, Aomori, 036-8216 Japan; Department of Radiation Oncology, Graduate School of Medicine, Hirosaki University, 5 Zaifu-cho, Hirosaki, Aomori, 036-8216 Japan; Department of Radiation Oncology, Graduate School of Medicine, Hirosaki University, 5 Zaifu-cho, Hirosaki, Aomori, 036-8216 Japan; Department of Radiation Oncology, Southern Tohoku BNCT Research Center, 7-10 Yatsuyamada, Koriyama, Fukushima, 963-8052 Japan; Department of Radiation Oncology, Graduate School of Medicine, Hirosaki University, 5 Zaifu-cho, Hirosaki, Aomori, 036-8216 Japan; Department of Radiation Oncology, Southern Tohoku BNCT Research Center, 7-10 Yatsuyamada, Koriyama, Fukushima, 963-8052 Japan; Department of Radiation Oncology, Graduate School of Medicine, Hirosaki University, 5 Zaifu-cho, Hirosaki, Aomori, 036-8216 Japan; Department of Radiation Oncology, Graduate School of Medicine, Hirosaki University, 5 Zaifu-cho, Hirosaki, Aomori, 036-8216 Japan; Department of Radiation Oncology, Graduate School of Medicine, Hirosaki University, 5 Zaifu-cho, Hirosaki, Aomori, 036-8216 Japan; Department of Radiation Oncology, Southern Tohoku BNCT Research Center, 7-10 Yatsuyamada, Koriyama, Fukushima, 963-8052 Japan; Department of Physical Therapy, Graduate School of Health Sciences, Hirosaki University, 66-1 Hon-cho, Hirosaki, Aomori, 036-8564 Japan; Department of Radiation Oncology, Graduate School of Medicine, Hirosaki University, 5 Zaifu-cho, Hirosaki, Aomori, 036-8216 Japan

**Keywords:** dual-energy CT, iodine density, lung cancer, stereotactic body radiotherapy

## Abstract

Lung cancer with low average iodine density measured via contrast-enhanced computed tomography (CT) using dual-energy CT technology has shown a reduced local control rate after stereotactic body radiotherapy (SBRT). The current study therefore investigated the relationship between low iodine density tumor area and its ratio and local recurrence after SBRT. Dual-energy CT was performed on the day before SBRT initiation, with a low iodine density tumor area being defined as that with an iodine density of <1.81 mg cm^–3^. The low iodine density tumor area, the ratio between the low iodine density tumor area and the entire tumor, and the local recurrence rate were then determined. No correlation was observed between the low iodine density tumor area and the local recurrence rate. However, tumors with a large low iodine density tumor area ratio showed an increased local recurrence rate, with the prognostic accuracy almost similar to that in previous studies using average iodine densities. Our results therefore suggest that the low iodine density tumor area ratio was a useful prognostic index after SBRT, with an accuracy comparable with that of the average iodine density.

## INTRODUCTION

Although patients with clinical stage I and II non-small cell lung cancer generally receive surgical treatment, many inoperable cases have been present due to advanced age or complications. As such, stereotactic body radiation therapy (SBRT) has been used as an alternative to surgery. While local control rates following SBRT have been similar to those after surgery in those with stage I and II lung cancer, local recurrence has been found in ~10% [1–4]. Factors predicting local recurrence after SBRT include tumor size [5, 6], histology [7], the maximum standardized uptake value (SUVmax) on fluorodeoxyglucose (FDG)-positron emission tomography (PET)/computed tomography (CT) [8, 9] and decreased tumor blood flow [10]. Although perfusion CT had previously been used to measure the decrease in tumor blood flow, dual-energy CT has recently allowed easy measurement of tumor blood flow. Studies have shown that gemstone spectral image analysis using dual-energy CT can measure material density [11, 12]. Moreover, dual-energy CT images generated from contrast-enhanced CT using an iodine contrast medium and measurement of tumor iodine density enable the evaluation of tumor blood volume. A comparison between the iodine density in the tumor and the local control rate measured using this technique found that lung cancer with low iodine density, indicating reduced blood flow, had increased local recurrence after SBRT [13]. These tumors with reduced blood flow and iodine density may contain a high percentage of hypoxic cells, which may lead to up-regulation of hypoxia-inducible factor 1 (HIF-1) and promote tumor growth [14, 15]. Given that HIF-1 up-regulation promotes tumor radioresistance, it has been considered a prognostic factor in non-small cell lung cancer [16].

As previous studies investigating the correlation between iodine density and prognosis have been based on only mean iodine density, no study has examined low iodine density tumor area or its ratio. Reports have shown that metabolic tumor volumes determined using FDG-PET/CT were a more accurate prognostic indicator than SUVmax and SUVmean after radiotherapy for lung cancer [17, 18]. When predicting prognosis using images, more accurate results are often obtained when evaluating tumor area with high malignancy and its ratio rather than single points, such as the maximum, minimum and average values. The current study therefore investigated the relationship between low iodine density tumor area and its ratio and prognosis after SBRT for early-stage lung cancer, and determined its usefulness as a prognostic indicator.

## MATERIALS AND METHODS

### Patient and tumor characteristics

The study was approved by the Institutional Review Board of our institution (Permission number: 2020-098). Informed consent was obtained in the form of opt out on the website. From March 2011 to December 2017, 151 patients (101 males and 50 females; median age 78 years; range 52–93 years) with 160 primary lung cancers who underwent dual-energy CT before SBRT were retrospectively reviewed. Among the included patients, 143 had one tumor, 5 had two tumors, 1 had three tumors and 2 had re-irradiated local recurrence after SBRT.

The clinical T stage was T1a to T2b based on the 8th edition of the Union for International Cancer Control (UICC) TNM 8 classification, while no lymph node and distant metastases were observed in all cases. A total of 14, 65, 49, 26 and 4 patients had T1a, T1b, T1c, T2a and T2b, respectively. The characteristics of the patients and tumors are summarized in Table 1.

**Table 1:** Characteristics of patients and tumors

Patient characteristics (*n* = 150)	
Age in years, median (range)	78 (52–93)
Sex (male/female)	101/50
Number of targets (1/2/3/re-irradiation)	143/5/3/2
Tumors (*n* = 160)	
Type (solid/part solid/GGO)	124/18/18
Clinical T (T1a/T1b/T1c/T2a/T2b)	14/65/49/26/4
Histology Adenocarcinoma Squamous cell carcinoma Large cell neuroendocrine carcinoma Unknown	81 33 4 45
Prescribed dose 50 Gy/5 fractions 60 Gy/6 fractions 54 Gy/9 fractions 56 Gy/8 fractions 60 Gy/10 fractions	125 30 1 1 3

GGO = ground-glass opacity.

### Treatment procedure

SBRT was performed using a plurality of fixed coplanar and non-coplanar beams on a 6 MV linear accelerator (Varian Medical Systems, USA). Patients were fixed using a custom-made headrest and immobilization system [19]. Optima (GE Healthcare, USA) was used for planning CT, with a slice thickness of 1.25 mm. When tumor movement under resting breathing was >1 cm, planning CT was performed by a breath-holding technique using Abches (APEX Medical Inc., Tokyo, Japan). When tumor movement under resting breathing was <1 cm, planning CT was performed with the 4D-CT technique using a real-time position management system (Varian Medical Systems, USA).

A 3D treatment planning system (XiO, version 4.8, ELEKTA, Stockholm, Sweden) was used for dose calculation with the following target settings. When 4D-CT was used for planning CT, the tumor range in all respiratory phases was set to gross tumor volume and internal target volume (GTV-ITV) delineated on CT images displayed with a window level of −300 Hounsfield units (HU) and a window width of 1700 HU. When the Abches was used for planning CT, the tumor range was set to GTV. The clinical target volume (CTV) was the GTV-ITV or the GTV plus a 3-mm margin in all directions, and the planning target volume (PTV) was the CTV plus a 5-mm margin in all directions. The 5-mm leaf margin was included around the PTV. An isocentric dose of 50 Gy was given for the PTV in five fractions for T1 tumors and of 60 Gy in six fractions for T2 tumors. For patients with tumors located near the organ at risk, the isocentric dose was changed to 54 Gy in nine fractions for one patient with a T1b tumor, to 56 Gy in eight fractions for one patient with a T1b tumor and to 60 Gy in 10 fractions for three patients with T1b/T2a/T2b tumors, respectively. A total dose was administered to the isocenter within a non-uniformity of 20% of the PTV dose. In most cases, the minimum dose to PTV corresponded to 85–95% of the prescribed dose. The median calculated biological effective dose, assuming an α/β ratio of 10 Gy (BED_10_), was 100 Gy (range 85.5–120 Gy).

**Fig. 1.**
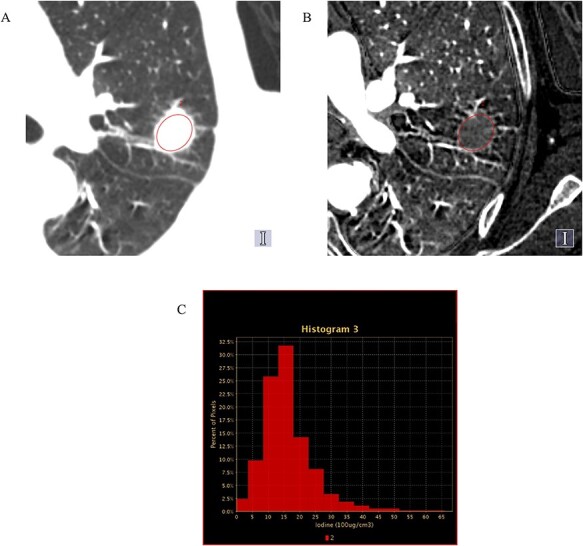
Location of the lung tumor regions of interest (red circle): computed tomography image of the pulmonary window (A); iodine (water) image (B). The iodine density inside the regions of interest is displayed as a histogram (C). In the histogram, the horizontal axis represents iodine density, while the vertical axis represents the ratio of the number of pixels.

### Scanning procedure

Dual-energy CT was performed using Discovery CT 750 HD (GE Healthcare, USA) with a fast kilovoltage- (kV) switching method on the day before treatment. Fast kV-switching CT utilizes a new garnet crystal scintillator detector with a rapid optical response and a high-voltage generator with an ultra-high-speed tube kV-switching mechanism. The non-ionic low osmolar contrast medium was administered at 600 mg I per kg of body weight, while iodine content was administered at 300 or 350 mg I per ml. The total amount of contrast medium was intravenously injected within 30 s. Scanning started at 25 s after injecting the contrast medium. The slice thickness of the image was 0.63 mm.

### Data analyses and considerations

All CT images were transferred to a workstation (GSI Viewer, GE Healthcare, USA) where the data were analyzed. The region of interest (ROI) was set on a single CT slice with the maximum cross-sectional diameter of the tumor. All of the following analyses are performed using the data inside the ROI set for this single CT slice. The ROI was set to surround the whole tumor as much as possible on the CT image in the lung window (window width 1000 HU; window level −700 HU) (Fig. 1A). The image was then converted to an iodine image, as shown in Fig. 1B. Then, we can see the number of pixels included in the ROI and the iodine density of each pixel. A histogram showing the ratio of the number of pixels for the iodine density of each pixel inside the ROI is presented in Fig. 1C. A specific iodine density was defined as the cut-off value, and the number of pixels with less than the cut-off iodine density value was counted, and this was defined as the low iodine density tumor area. The ratio of the low iodine density tumor area to the total number of pixels contained in the ROI was defined as the low iodine density tumor area ratio. The median average iodine density of all tumors was used as the cut-off value, after which the tumor area with less than the cut-off iodine density value was calculated from this histogram and defined as the low iodine density tumor area. The ratio of the low iodine density tumor area to the ROI was defined as the low iodine density tumor area ratio.

### Follow-up and statistical analysis

The main endpoint of this study was local recurrence rates, which were calculated starting from treatment onset until local recurrence. Local recurrence was diagnosed with local tumor enlargement on CT, which continued for 6 months or more. Cases suspected of local recurrence underwent FDG-PET/CT and/or tissue biopsy when possible, although this was not essential. The relationship between low iodine density tumor area and its ratio and local recurrence rates after SBRT was investigated by a competing risk analysis to adjust for death from other causes. Local recurrence after SBRT and death from other causes were two event types in this analysis. We used the cumulative incident function (CIF) to describe the probability of local recurrence, and Gray’s test to compare CIF between categories. The Pearson product–moment correlation coefficient (*r*) was used to determine the relationship between maximum diameter of the tumor and low iodine density tumor area and its ratio. The prognostic accuracy of the average tumor iodine density and low iodine density tumor area or ratio was compared using the receiver operating characteristic (ROC) curve. Competing risk analysis and Gray’s test were performed using R version 4.0.2. ROC curves were performed using the IBM SPSS statistics version 22.0 software package (SPSS Inc., Chicago, IL, USA). *P* values <0.05 indicated statistically significant differences.

## RESULTS

### Treatment outcomes

**Fig. 2.**
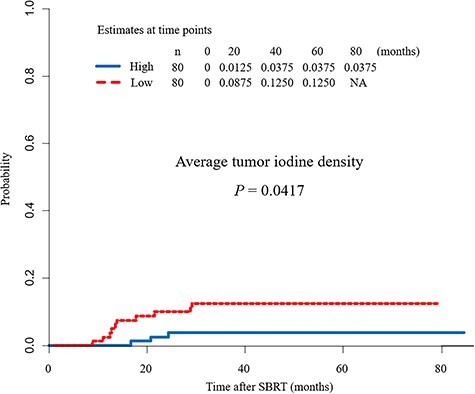
Comparison of the local recurrence rate according to average tumor iodine density. The low iodine density group had a significantly higher local recurrence rate than the high iodine density group (*P* = 0.0417). The 3-year local recurrence rates were 3.7% and 12.5% in the high and low groups, respectively.

The median observation period was 25.7 months. Of the 160 tumors, 13 had local recurrences and 28 died from other causes. The 5-year CIF for local recurrence and competing death were 8.1% [95% confidence interval (CI) 4.5–13.0%] and 15.6% (95% CI 10.4–21.7%), respectively. The median average tumor iodine density was 1.81 mg cm^–3^ (range 0.15–5.37 mg cm^–3^). The median of GTV was 5.65 cm^3^ (range 0.67–45.0 cm^3^). Local recurrence rates were compared by dividing the patients into two groups according to average tumor iodine density (i.e. a high and low value group), with the cut-off value set at the median average tumor iodine density of 1.81 mg cm^–3^. Accordingly, the low iodine density group had significantly higher local recurrence rates than the high iodine density group (*P* = 0.0417), with the 3-year local recurrence rates being 3.7% and 12.5% in the high and low groups, respectively (Fig. 2).

### Evaluation of the low iodine density tumor area

**Fig. 3.**
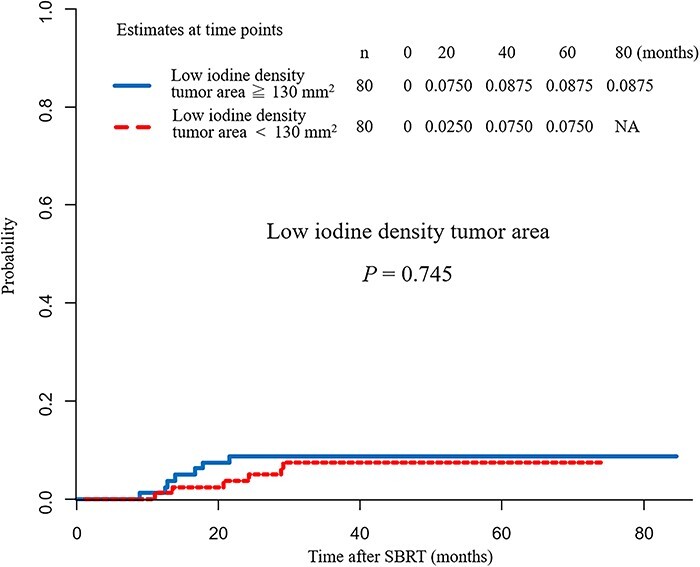
Comparison of the local recurrence rate according to the low iodine density tumor area. No significant difference was observed in the local recurrence rate between the two groups divided by the median low iodine density tumor area (cut-off value of 130 mm^2^) (*P* = 0.745).

The median average iodine density for all tumors was 1.81 mg cm^–3^, which was set as the cut-off value. Tumor areas with an iodine density of <1.81 mg cm^–3^ were defined as the low iodine density tumor areas. After evaluating the correlation between low iodine density tumor area and the local recurrence rate, no significant difference was found in local recurrence rates according to low iodine density tumor area, with a cut-off median low iodine density tumor area of 130 mm^2^ (*P* = 0.745) (Fig. 3).

### Evaluation of the low iodine density tumor area ratio

**Fig. 4.**
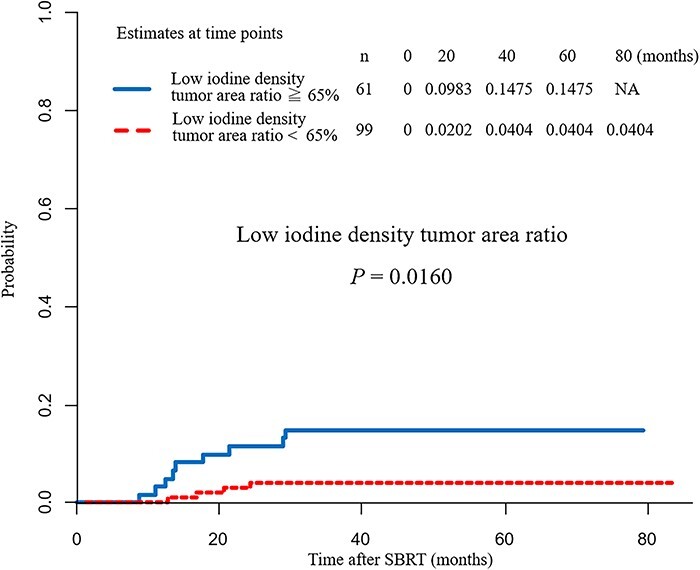
Comparison of local recurrence rates with a low iodine density tumor area ratio cut-off of 65%. The local recurrence rate showed the largest difference when the low iodine density tumor area ratio cut-off was set to 65% (*P* = 0.0160). The 3-year local recurrence rate was 4.0% and 14.7% in the small and large low iodine density tumor area ratio group, respectively.

The ratio of the low iodine density tumor area to the ROI was defined as the low iodine density tumor area ratio. To examine the low iodine density tumor area ratio most closely related to the local recurrence rate, we compared local recurrence rates for every 5% increment in low iodine density area ratio from 10% to 90%. Accordingly, the local recurrence rate showed the largest difference at a low iodine density tumor area ratio cut-off of 65% (*P* = 0.0160) (Fig. 4), with a 3-year local recurrence rate of 4.0% and 14.7% in the small and large low iodine density tumor area ratio groups, respectively (Fig. 4).

In addition, the local recurrence rate for each low iodine density tumor area ratio is shown in Table 2. Tumors with a low iodine density tumor area ratio of 60% or more tend to have a high local recurrence rate.

**Table 2:** Local recurrence rate for each low iodine density tumor area ratio

Low iodine density tumor area ratio (%)	Number of tumors (*n* = 160)	Number of local recurrences (*n* = 13)	Local recurrence rate (%)
0–10	10	1	10.0
10–20	9	0	0
20–30	18	0	0
30–40	16	2	12.5
40–50	21	0	0
50–60	19	1	5.3
60–70	17	2	11.8
70–80	26	4	15.4
80–-90	13	2	15.4
90–100	11	1	9.1

### Comparison of prognostic accuracy between average tumor iodine density and low iodine density tumor area ratio

**Fig. 5.**
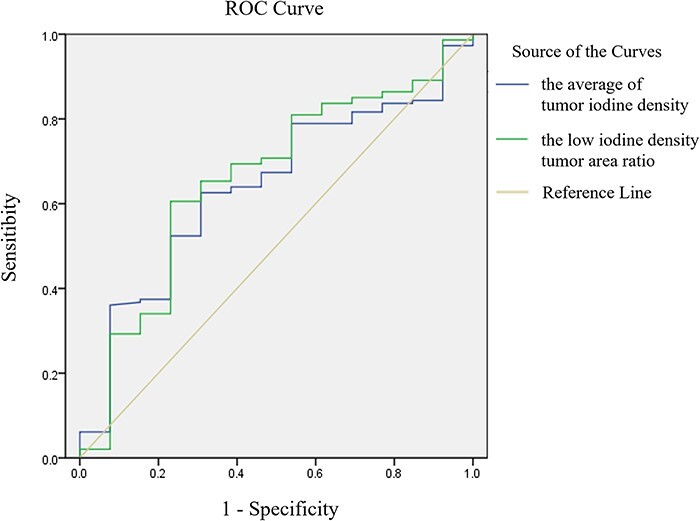
Receiver operating characteristic (ROC) curves of the average tumor iodine density and low iodine density tumor area ratio. The prognostic accuracy was almost the same with both predictors, but was slightly higher with the low iodine density tumor area ratio.

Prognostic accuracy of the average tumor iodine density and the low iodine density tumor area ratio was compared using the ROC curve. Accordingly, the area under the curve was 0.639 and 0.658 for the average tumor iodine density and low iodine density tumor area ratio, respectively. Although prognostic accuracy was almost the same with both predictors, it was slightly higher with the low iodine density tumor area ratio (Fig. 5).

### Correlation between maximum tumor diameter, average tumor iodine density, low iodine density tumor area and its ratio

The maximum diameter of the tumors was positively correlated with low iodine density tumor area (*P* < 0.001, *r* = 0.69), as well as low iodine density tumor area ratio (*P* < 0.001, *r* = 0.31). Low iodine density tumor area was more strongly correlated with the maximum diameter of the tumor.

Average tumor iodine density was negatively correlated with low iodine density tumor area (*P* < 0.001, *r* = −0.52), as well as low iodine density tumor area ratio (*P* < 0.001, *r* = −0.89). Low iodine density tumor area ratio was more strongly correlated with average tumor iodine density.

### Balancing difference in iodine density due to cardiac output

To balance differences in cardiac output, the relationship between the local recurrence rate and the value obtained by dividing the tumor iodine density by the aortic iodine density was examined. Accordingly, the median tumor iodine density/aortic iodine density value was 0.135, with no significant difference in local recurrence rate observed between patients having higher and lower values (*P* = 0.1522).

## DISCUSSION

The current study found that a low iodine density tumor area ratio can be a useful prognostic indicator after SBRT for lung cancer. Only a few studies have investigated the relationship between tumor iodine density measured using dual-energy CT and the local control rate after SBRT for early lung cancer. To our knowledge, the present study has been the first to examine the correlation between the local recurrence rate and low iodine density tumor area and its ratio.

The local recurrence rate after SBRT in this study was comparable with that presented in previous reports [1–4]. A comparison of average tumor iodine density showed a higher local recurrence rate in tumors with a lower average tumor iodine density, that is comparable with that in a previous report [13].

We found a positive correlation between maximum diameter of the tumor and low iodine density tumor area and its ratio, with the correlation between maximum diameter of the tumor and low iodine density tumor area being particularly strong. Reports have shown that local recurrence rates after SBRT increase as the maximum diameter of the tumor increases [19]. Given that a low iodine density tumor area was strongly related to the maximum diameter of the tumor, it may seem to be a prognostic indicator of the local recurrence rate. However, no relationship between a low iodine density tumor area and the local recurrence rate had been observed. Instead, the low iodine density tumor area ratio was correlated with the prognosis of local recurrence. After comparing the prognostic accuracy of the low iodine density tumor area ratio and average tumor iodine density using ROC curves, our analysis showed similar prognostic accuracy.

Iodine density measured using dual-energy CT has been considered to reflect blood flow. Conventionally, perfusion CT has been used to measure tumor blood flow. Animal experiments have shown that microvascular density (an index for quantifying tumor angiogenesis), tumor blood flow biomarkers [such as vascular endothelial growth factor (VEGF)] and tumor blood flow measured using perfusion CT were positively correlated [20]. Tumors with reduced blood flow become hypoxic and adapt to such conditions by performing anaerobic glycolysis through the up-regulation of the transcription factor HIF-1 [21]. Given that HIF-1 up-regulation promotes tumor radioresistance, it has been considered a prognostic factor in non-small cell lung cancer [16].

Given its strong correlation with the maximum diameter of the tumor, a known prognostic factor, a low iodine density tumor area has been considered a prognostic indicator of local recurrence rate. Unexpectedly, however, our results showed no such relationship with the local recurrence rate. One hypothesis for this unexpected result may be the influence of reoxygenation. Tumors obtain oxygen from capillary vessels within a radius of 100–180 μm, while tumor cells existing further away are chronically hypoxic. In fractionated radiation therapy, most of the tumor oxic cells near the capillaries die after the first dose. When tumor cells near the capillaries become necrotic, oxygen is supplied to the tumor cells distant from the capillaries, reoxygenating the hypoxic cells. Reoxygenated tumor cells then become radiosensitive and die upon subsequent irradiation. The hypoxic tumor cells that were radioresistant before treatment initiation can be gradually reduced and completely eradicated by repeating this process. Tumors with a decreased low iodine density tumor area ratio have a higher proportion of oxygenated tumor cells that die in the early stages of irradiation. Thus, hypoxic tumor cells located farther from the capillaries are more likely to be reoxygenated and become more radiosensitive. On the other hand, tumors with an increased low iodine density tumor area ratio have a lower proportion of oxygenated tumor cells that die in the early stages of irradiation. Thus, hypoxic tumor cells located farther from the capillaries are less likely to be reoxygenated and remain radioresistant, which increases the local recurrence rate. For this reason, a correlation was observed between low iodine density tumor area proportion and prognosis.

From this result, it may seem that the low iodine density tumor area ratio and the average tumor iodine density are the same indicators. Most tumors have one peak of iodine density, as shown in the histogram in Fig. 1C, but some tumors have two peaks of iodine density. For tumors with two peaks of iodine density, the average tumor iodine density does not always reflect the amount or proportion of low iodine density tumor. For these tumors, the low iodine density tumor area ratio may be a more accurate indicator of prognosis after radiation therapy, rather than the average tumor iodine density.

The average tumor iodine density used in previous studies and the low iodine density area ratio used in the present study showed similar prognostic accuracy following SBRT for early stage lung cancer. Low iodine density was defined as that below 1.81 mg cm^–3^, which was the median average tumor iodine density obtained in this study. However, considering that a more optimal cut-off value may perhaps exist, further investigations are required. Surgery or increased treatment intensity through increased total dose or combined chemotherapy may be needed for tumors with an increased low iodine density tumor area ratio.

### Limitations

Several factors could influence tumor iodine density. Firstly, a low iodine density tumor area ratio should be measured by volume. However, the workstations used in our institution cannot evaluate iodine density by volume. Therefore, we evaluated the low iodine density tumor area and its ratio on a single CT slice. A study that evaluated the low iodine density tumor area ratio by volume should be added. Secondly, depending on the shape of the tumor, it was difficult for the ROI to completely cover the whole tumor. This may affect the value of tumor iodine density. Other factors such as scan delay, total amount of contrast agent, infusion rate and patient characteristics could influence tumor iodine density.

To balance differences in cardiac ejection fraction, the relationship between the local control rate and the value obtained by dividing the tumor iodine density by the aortic iodine density was examined. Notably, no relationship was observed between both values. Lung cancer involves both the bronchial and pulmonary arteries, and increases pulmonary artery perfusion [22]. Therefore, it may be inappropriate to balance differences in cardiac ejection fraction by using aortic iodine density that reflects bronchial artery blood flow. Given that dual-energy CT provides images obtained at a temporary point, determining blood volume flowing into the tumor is difficult. Thus, establishing methods for correcting differences in the contrast medium arrival state during imaging due to cardiac ejection fraction is necessary.

## CONCLUSION

The present study showed that a low iodine density tumor area ratio is a useful prognostic index following SBRT. Iodine density measurements using dual-energy CT can therefore be a quantitative evaluation of tumor blood flow and can be a simple evaluation index of radioresistance.

## Conflict of interest

The authors declare that there are no conflicts of interest.

## Funding

This work was supported by Japan Society for the Promotion Science (JSPS) KAKENHI grant number 20 K16749.
